# Sex-specific components of frailty in C57BL/6 mice

**DOI:** 10.18632/aging.102114

**Published:** 2019-07-29

**Authors:** Cory W. Baumann, Dongmin Kwak, LaDora V. Thompson

**Affiliations:** 1 Divisions of Rehabilitation Science and Physical Therapy, Department of Rehabilitation Medicine, Medical School, University of Minnesota, Minneapolis, MN 55455, USA; 2 Department of Physical Therapy and Athletic Training, Boston University, Boston, MA 02215, USA

**Keywords:** frailty, sex differences, C57BL/6 mice, frailty phenotype, health-survival paradox

## Abstract

Many age-related biochemical, physiological and behavioral changes are known to be sex-specific. However, how sex influences frailty status and mortality risk in frail rodents has yet to be established. The purpose of this study was therefore to characterize sex differences in frail mice across the lifespan. Male (n=29) and female (n=27) mice starting at 17 months of age were assessed using a frailty phenotype adjusted according to sex, which included body weight, walking speed, strength, endurance and physical activity. Regardless of sex, frail mice were phenotypically dysfunctional compared to age-matched non-frail mice, while non-frail females generally possessed a higher body fat percentage and were more physically active than non-frail males (p≤0.05). The prevalence of frailty was greater in female mice at 26 months of age (p=0.05), but if normalized to mean lifespan, no sex differences remained. No differences were detected in the rate of death or mean lifespan between frail male and female mice (p≥0.12). In closing, these data indicate that sexual differences exist in aging C57BL/6 mice and if the frailty criteria are adjusted according to sex, the prevalence of frailty increases across age with frail mice dying early in life, regardless of sex.

## Introduction

Aging is characterized by a gradual decline in various health parameters across multiple biochemical, physiological and behavioral systems [1]. With dysfunction of these systems, quality of life is impaired (i.e., reduced healthspan), which ultimately leads to death. Interestingly, a subset of aging individuals appears to lack resilience within these systems, a condition termed frailty. Frailty has broadly been defined as an age-associated biological syndrome characterized by an exaggerated vulnerability to adverse global health outcomes, a reduced capacity to react to stressors and an overall loss in physiological function [2,3]. Consequently, frail individuals are at a greater risk of falls, dependency, disability, institutionalization, hospitalization and mortality [4,5]. Studying frailty has therefore become a pivotal area of research.

Questions in the field of frailty have largely gone unanswered due to the inherent limitations of clinical studies, including biological, ethical and logistical complications of working with older individuals [6,7]. The recent development of assessment tools for frailty in mice reverse-translated from human frailty models has made it possible to bypass many of these aforementioned limitations [8]. For instance, Howlett and colleagues [7] established a mouse frailty index based on accumulated deficits derived from Rockwood et al. [9,10], that assessed 31 invasive and noninvasive variables (i.e., activity levels, hemodynamic measures, body composition and basic metabolic status), which was later simplified to only include readily apparent noninvasive signs of clinical deterioration [11]. Similarly, Thompson and colleagues [8] developed a frailty phenotype in aged mice that was similar to the clinical criteria used by Fried et al. [5], which included grip strength, walking speed, physical activity and endurance. They recently modified the phenotype to also include body weight [12,13]. The development of rodent frailty assessment tools represents an important step forward in how we understand frailty, and interventions that may delay or prevent its progression.

Recent efforts to understand frailty have extended beyond this biological syndrome solely being age-dependent, but rather age- and sex-dependent. Many age-related biochemical, physiological and behavioral changes are already known to be sex-specific [14–17], which gives credence to the premise that sex may also be an important variable associated with frailty status. In fact, women have higher frailty index scores than men at all ages but paradoxically have a lower mortality risk, a concept termed the sex-frailty paradox [18,19]. A phenomenon also known more broadly as the male-female health-survival paradox; women have poorer health than men, characterized by greater burden of chronic disease, more disability and worse self-rated health, but live longer [20,21]. These data suggest each sex may have different physiological reserves, and that the link between health status and mortality is not necessarily fixed in humans. To date, very little is known regarding sex differences in frail mice or if the sex-frailty paradox extends beyond humans. The purposes of this study were therefore to characterize sex differences in frail C57BL/6 mice across the lifespan using a validated frailty phenotype, and assess how closely it relates to the human sex-frailty paradox.

## RESULTS

**Figure 1:**
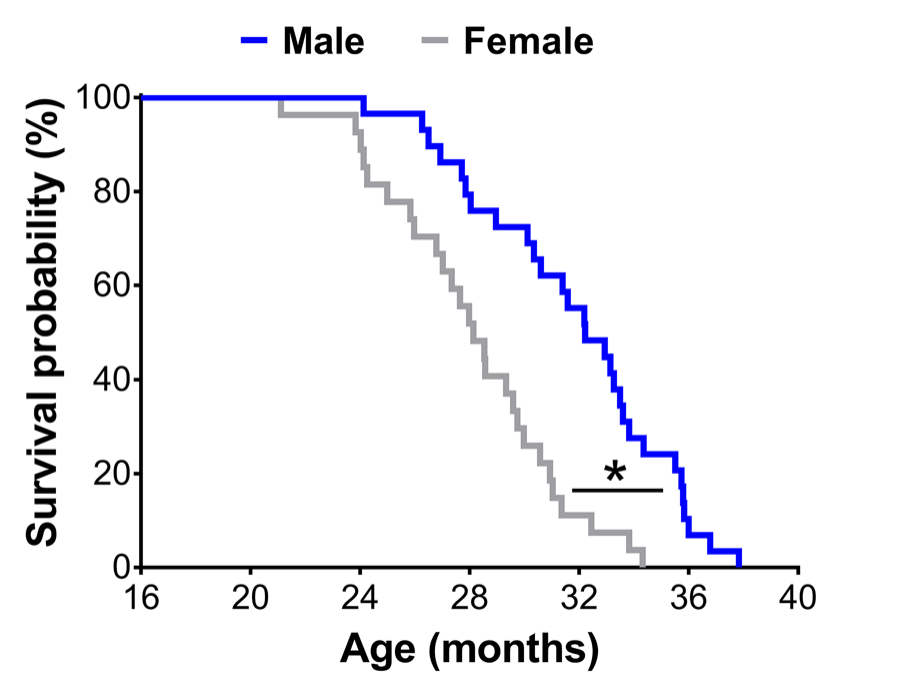
Survival curves of male (n=29) and female (n=27) C57BL/6 mice in the original cohort. * Significant difference between sex (p≤0.05).

A Kaplan-Meier survival analysis revealed male mice lived 13% longer than female mice (mean lifespan: 31.8±0.7 vs. 28.1±0.6 months; p<0.01) (Figure 1).

**Figure 2:**
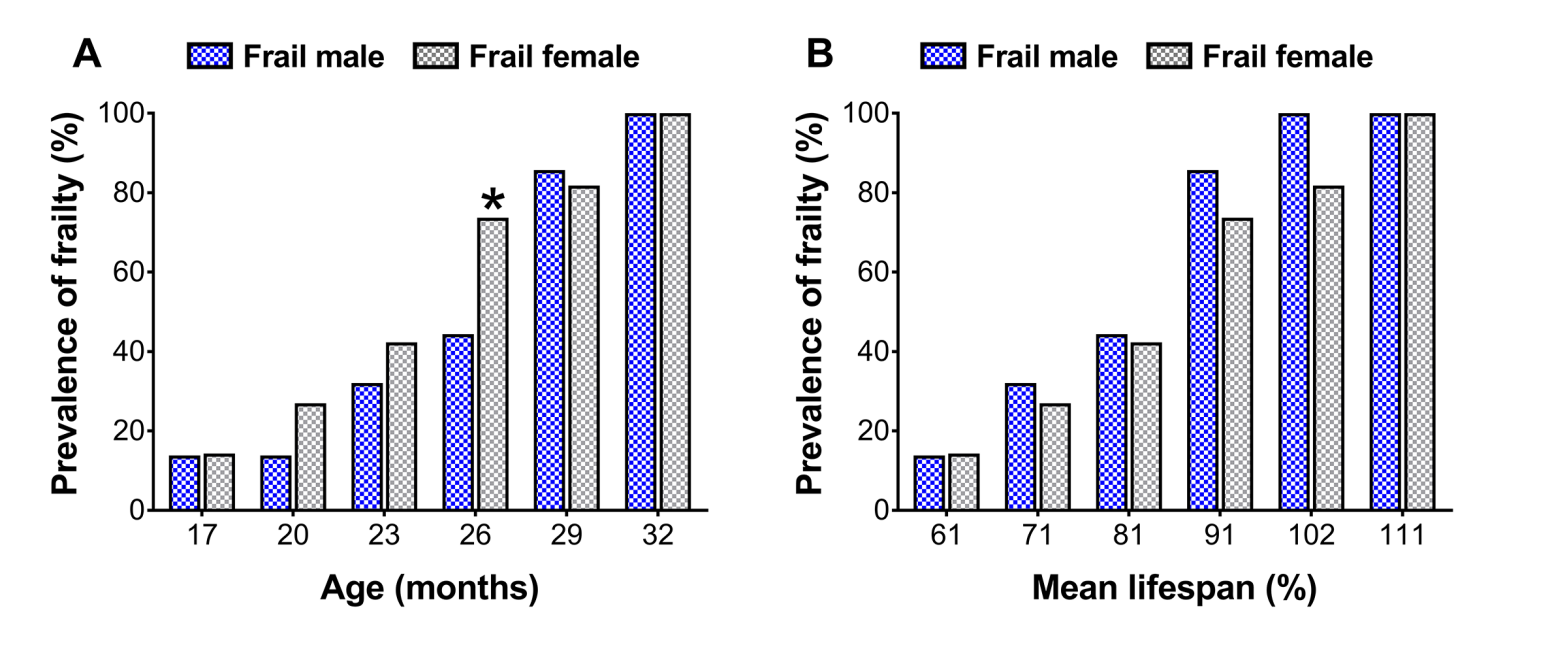
Prevalence of frailty in male and female mice at each time-point (**A**) and normalized to the mean lifespan for each respective sex (**B**). The number of frail mice varies by time-point (n=3-18). * Significant difference between sex (p≤0.05).

To assess sex differences in the prevalence of frailty across the lifespan, cut-off values determined at 23 and 20 months for males and females (Table 1, also see ‘Frailty criteria’ in the METHODS), respectively, were used to categorize mouse frailty from 17 months until death [12,13]. There were more frail female mice at 26 months than frail male mice (44.4 vs. 73.7%; p=0.05) (Figure 2A), however when normalized to the mean lifespan for each sex, no differences were detected (p≥0.10) (Figure 2B).

**Table 1:** Frailty criteria and cut-off values in male and female C57BL/6 mice.

Human frailty phenotype Fried et al. [5]	Mouse frailty phenotype	Cut-off (%)	Mouse cut-off values	
			Male (23 months) Baumann et al. [12]	Female (20 months) Kwak et al. [13]
Low activity	Voluntary wheel running	Lower 20%	1.088 km/day	1.249 km/day
Poor endurance	Treadmill fatigue test	Lower 20%	944.2 sec	915.6 sec
Weakness	Grip meter	Lower 20%	220.3 g	239.1 g
Slowness	Rotarod test	Lower 20%	38.0 sec	27.6 sec
Lower body weight	Body weight	Upper 20%	40.6 g	44.1 g

**Figure 3:**
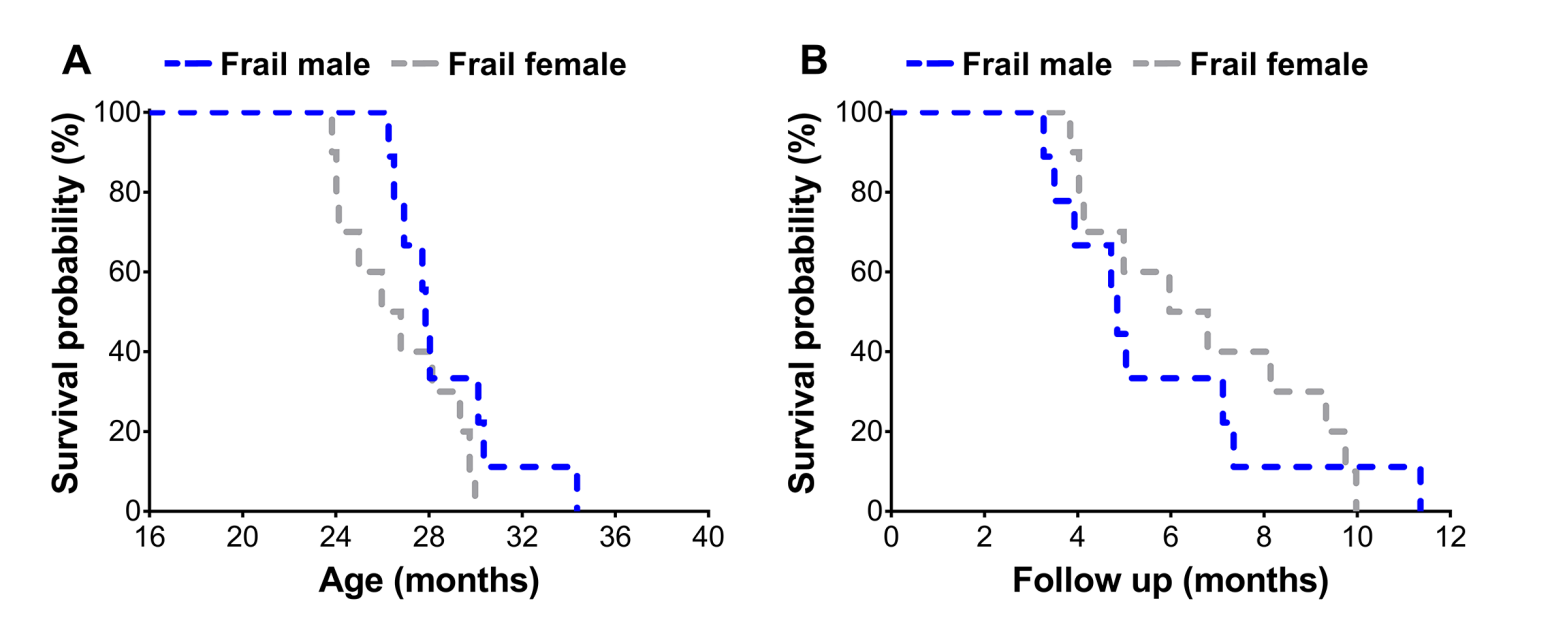
Survival curves of frail male (n=9) and female (n=10) mice. Mice were classified as frail according to the frailty criteria listed in Table 1, and survival was assessed across the lifespan (**A**) and also relative to the time of the frailty assessment (**B**).

Frail mice were then assessed using a Kaplan-Meier survival analysis to determine sex differences on mortality risk (Figure 3A & B). Frail female mice (n=10) had a mean survival time of 26.7±0.8 months, which was not different (p=0.12) from the mean survival time of 28.7±0.9 months observed in frail male mice (n=9) (Figure 3A). There was also no difference between mortality rate of frail male and female mice when evaluated by survival following the frailty assessment (p=0.78) (Figure 3B). See ‘Frailty criteria’ in the METHODS for details on the frailty assessment components.

**Figure 4:**
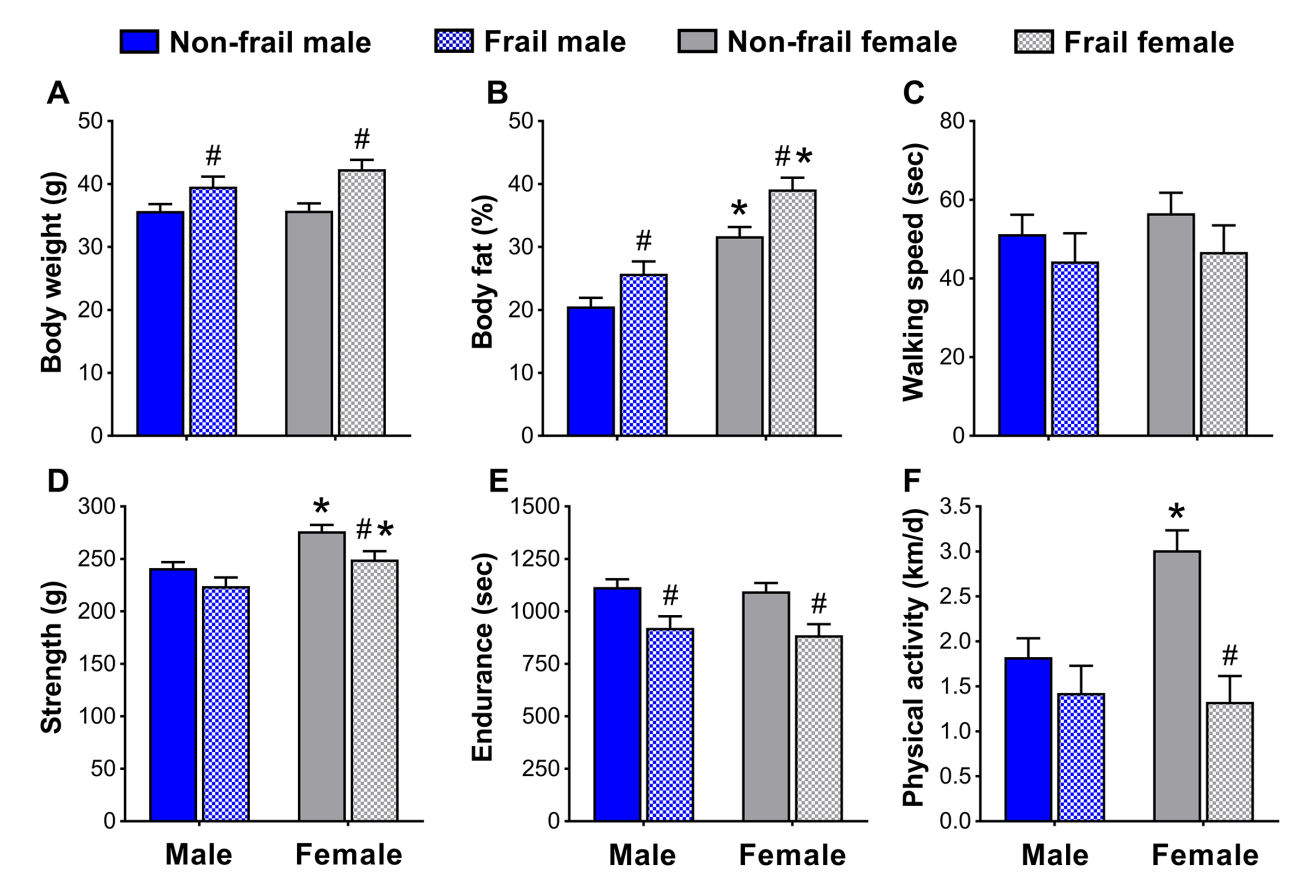
Body composition (**A, B**) and physical function (**C-F**) of non-frail and frail, male and female mice at the time of the frailty assessment. *Significant difference between sex (p≤0.05). **^#^**Significant difference between frailty status within sex (i.e., non-frail vs. frail male, non-frail vs. frail female) (p≤0.05). Values are presented as mean + standard error.

**Figure 5:**
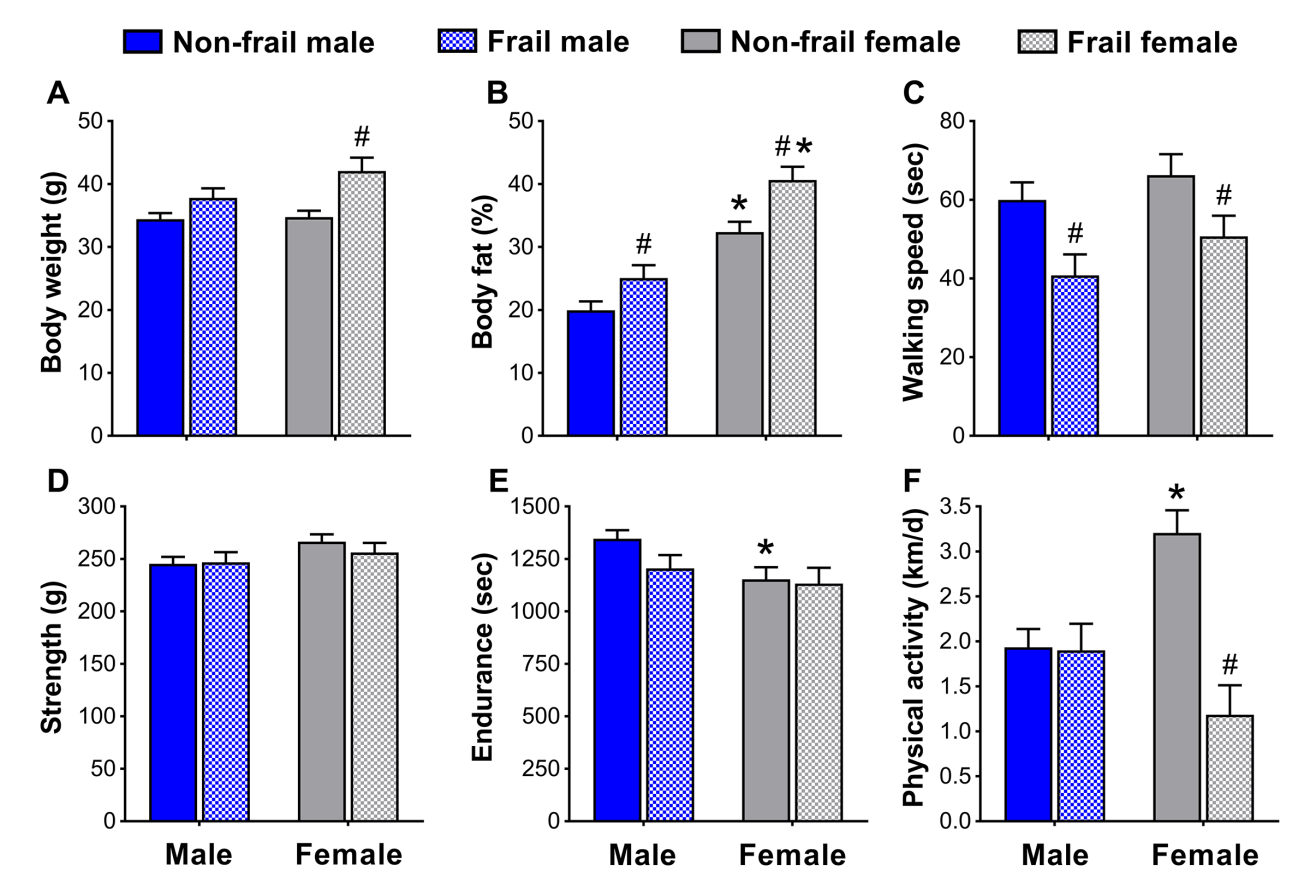
Body composition (**A, B**) and physical function (**C-F**) of non-frail and frail, male and female mice three months prior to the frailty assessment. *Significant difference between sex (p≤0.05). #Significant difference between frailty status within sex (i.e., non-frail vs. frail male, non-frail vs. frail female) (p≤0.05). Values are presented as mean + standard error.

To identify if non-frail and frail mice presented anthropometrical and/or functional sex differences early in life, male and female mice were compared at the time of the frailty assessment and three months prior. Frail mice, regardless of sex, were heavier (p≤0.05) (Figure 4A and 5A) and possessed more body fat (p≤0.05) (Figure 4B and 5B) than non-frail mice. However, non-frail and frail female mice had 53.1-62.2% more body fat compared to non-frail and frail male mice (p≤0.01) (Figure 4B and 5B).

As with body weight and body fat percentage, time to fatigue (i.e., endurance, Figure 4E) at the frailty assessment and time on the rotarod (i.e., walking speed, Figure 5C) in the months prior were less (p≤0.05) for frail mice compared to non-frail mice. Frail females were weaker than non-frail females (p≤0.05), but both were stronger than males at the time of the frailty assessment (p≤0.03) (Figure 4D). In the months prior, non-frail males had better endurance scores compared to non-frail females (p=0.01) (Figure 5E). Physical activity measured by voluntary wheel run distance was 65.2-65.7% greater in non-frail females compared to non-frail males, and 127.1-170.8% greater than that of frail females (p≤0.01) (Figure 4F and 5F).

## DISCUSSION

The purposes of this study were to characterize sex differences of frail mice across the lifespan using a validated frailty phenotype, and assess how closely it relates to the human sex-frailty paradox. Our results generated three primary findings. First, the prevalence of frailty was greater in female mice but was age-dependent, and if normalized to mean lifespan, no sex differences remained. Second, frail mice, regardless of sex, died at a similar rate after the frailty assessment and had comparable mean lifespans. Lastly, anthropometrical and functional sex differences impact the selection of frailty criteria and determination of cut-off values. These results do not fully support the sex-frailty paradox observed in humans, but do suggest that regardless of sex, frail C57BL/6 mice are anthropometrically and functionally dysfunctional, and die early in life [12,13].

Women experience greater levels of frailty, which is associated with poor health and an increased risk of death, but paradoxically live longer than men [18,19]. This phenomenon has been termed the male-female health-survival paradox [20,21], also coined the sex-frailty paradox [18,22]. To date, it is currently unknown if this paradox extends to common rodent models (e.g., C57BL/6 mice) used to study frailty and aging. For comparison between human and rodents, we have outlined the sex-frailty paradox into two components: 1) the prevalence of frailty and 2) the mortality risk of those identified as frail.

The first component of the sex-frailty paradox in humans is that frailty scores are greater in women. In the laboratory, this component has not been fully established in mice. In fact, some report no sex differences [7,23] while others report greater frailty scores in female C57BL/6 mice [11], and more recently in female NIH Swiss mice [24]. Our data partially support the latter findings that female mice exhibit greater levels of frailty, though it was age-dependent and only transient. Using a longitudinal lifespan study design with a phenotypic frailty assessment tool, we observed that at 26 months of age the prevalence of frailty was 66.0% greater in females than males (73.7 vs. 44.4%), however no differences were detected at any other age (Figure 2A). Furthermore, the prevalence of frailty in males increased to a similar extent from 26 to 29 months as that seen in females from 23 to 26 months. Aging female NIH Swiss mice also experience a sharp increase in frailty scores beyond that of males when assessed using a physiological frailty index [24]. It is possible that these observations [present study, 24] are linked to hormones, immune function or regional distribution of body fat, factors associated with sex differences in response to life-extending genetic or pharmacological interventions [25]. Together, these data partially support that the prevalence of frailty is greater in females but is likely age-dependent, as all mice (regardless of sex) eventually become frail.

The observation that female mice have greater frailty scores or prevalence of frailty is not a certainty and with further interpretation, can be challenged. Indeed, in the present study male mice lived 13% longer than female mice (Figure 1). Several other groups have shown C57BL/6 male mice live longer than female mice, though there is a fair amount of variability [26,27]. Austad and Fischer [28] recently reviewed 29 studies on the C57BL/6 mouse strain and found that males lived longer in 18 studies and females lived longer in 11 studies. Therefore, it is critical researchers consider sex differences in lifespan and make the proper adjustments to their frailty scores and cut-off values. In the present study, frailty cut-off values were selected at different time-points based on the 3.7-month variance in mean lifespan between sex, with frailty determination occurring at 23 and 20 months for male and female mice, respectively (Table 1). Taking this rationale a step further, we normalized the prevalence of frailty in male and female mice to the mean lifespan for each sex, which therefore takes into account the three month offset previously mentioned (i.e., 23 vs. 20 months). Using this approach, the sex difference observed in the prevalence of frailty was eliminated, indicating that the prevalence of frailty was similar between male and female C57BL/6 mice (Figure 2B). These results and their subsequent interpretations suggest that sex differences in lifespan be taken into account when comparing male and female rodents.

The second component of the sex-frailty paradox is that women live longer than men despite having higher frailty scores [18,19]. Our data does indicate that at one time-point (without normalization to the mean lifespan), the prevalence of frailty was greater in females (Figure 2A). However, when assessing mortality risk of frail mice across sex, we did not observe that frail female mice lived any longer. In fact, regardless of sex, frail mice died at a similar rate following the frailty assessment at 23 and 20 months for male and female mice, respectively (Figure 3A & 3B). Approximately 50% of the frail male and female mice died within six months after being identified as frail (Figure 3B). Our data suggest frail male and female C57BL/6 mice die at a similar rate when frailty cut-off values are adjusted to reflect variability in sex and lifespan. Overall, in our cohort of aging C57BL/6 mice, it appears frail females do not paradoxically live longer than frail males; and therefore, does not support the second component of the sex-frailty paradox observed in humans.

After determining the prevalence of frailty and overall mortality risk did not appear to have prominent sex differences, we turned to examining sex-specific characteristics used in the frailty assessment tool. Kane et al. [29] recently articulated the concept that, “there are different ways to become frail and each of them is valid for the person or mouse who becomes frail in that way,” a concept we explored further and assessed whether it was also sex-specific. Our results indicate that although some frailty markers were similar between sex, others appeared to be dependent on being male or female. For instance, non-frail female mice were more physically active than non-frail males, a common sex difference observed in mice [30,31]. It is therefore important to acknowledge that some sex differences will be present when determining frailty criteria and cut-off values. The observation that non-frail females ran ~65% further on the voluntary wheels than non-frail males is a prime example of a functional sex difference (Figure 4F & 5F), and solid justification that frailty cut-off values cannot simply be applied across sex. Fried’s human phenotype [5] acknowledged this by using cut-off percentiles according to gender and Antoch et al. [24] alluded to this in mice, by reporting parameters of their physiological frailty assessment, which only showed partial overlap between males and females. We suggest that sex differences be considered when assessing frailty, and emphasize that there are different ways to become frail – one of which may be sex.

In closing, using a longitudinal lifespan study design we characterized sex differences in frail C57BL/6 mice via a validated frailty phenotype, and compared our findings to that of the sex-frailty paradox observed in humans. Our results suggest phenotypic sex differences exist in C57BL/6 mice and that males live longer than females. Researchers should therefore be cognizant of the anthropometrical, functional and lifespan differences between males and females, and adjust frailty criteria based on sex and mean lifespan – as done in the present study. Despite these sex differences, the prevalence of frailty was generally the same between sexes, with the exception of a single age when frailty was greater in females. Moreover, after a mouse was identified as frail, male and female mice died at a similar rate. Together, these results (i.e., in C57BL/6 mice) do not fully support the sex-frailty paradox observed in humans. In closing, these data indicate that sex differences exist in aging C57BL/6 mice and if the frailty criteria are adjusted accordingly, the prevalence of frailty increases across age with frail mice dying early in life [12,13], regardless of sex.

## METHODS

### Ethical approval and animals

Male (n=29) [12] and female (n=27) [13] C57BL/6 mice were purchased from Jackson Laboratory (Bar Harbor, ME, USA) at 13 and 5 months, respectively. Mice were housed under a 12 hour light:dark cycle at 20-23^o^C in specific pathogen-free facilities, supplied with food and water *ad libitum*, and allowed to age and die of natural causes. All animal procedures were in accordance with the standards set by the Institutional Animal Care and Use Committees at the University of Minnesota and Boston University.

### Experimental design

Testing was initiated at 17 months of age and continued every 3 months (20, 23,... 32 or death) making this study a repeated measures research design, which allowed us to determine the lifespan of each individual mouse. For each performance testing period, mice were subjected to a battery of assessments over a one-week period during every three-month interval, as previously used by Thompson and colleagues [12,13]. All testing procedures followed the same protocol during each assessment, and to ensure testing reliability, all assessments were completed by the same testers.

### Body weight and body fat percentage

Body weights were obtained on an electronic scale (CS-200, Ohaus, Parsippany, NJ, USA), while body fat percentage was evaluated using a Lunar PIXImus densitometer (GE Lunar Corporation, Madison, WI, USA). Briefly, a phantom mouse was first used as a calibration standard for quality control prior to each testing day. Mice were then anesthetized with isoflurane, placed on an adhesive specimen tray, and scanned with the skull excluded and tail included.

### Walking speed

Walking speed was evaluated using a rotarod (Rota-Rod R/S; LSi Letica, Cornella, Spain). Mice were first warmed-up on the rotarod by walking at 4 rpm for 30 seconds, at which point rotarod speed increased 1 rpm every 8 seconds up to 40 rpm over a 5 min period. Walking speed was recorded when the mouse was unable to sustain the rotation speed of the rotarod. Each mouse performed three trials with a 10-minute rest period in-between each trial. The best score of these trials, recorded as seconds, was used as walking speed.

### Strength

Strength was evaluated using a grip meter test (P/N760483, Coulbourn Instruments, Whitehall PA). Mice were gently lowered over the top of a wire grid so that the front and hind paws gripped the grid. Once gripped, the tail of each mouse was pulled back steadily, keeping the mouse’s torso in a horizontal position. When the mouse was unable to maintain its grip, the trial was over and the grip strength, in grams, was recorded. Each mouse performed two trials with a 10-minute rest period in-between each trial. The best score of these trials was used as peak grip strength.

### Endurance

Endurance was evaluated using a time to fatigue test on a motorized treadmill (Exer 3/6 Treadmill; Columbus Instruments, Columbus, OH). After a brief warm-up (5 m/min for 5 min), mice remained on the treadmill and time to fatigue began with speed increasing 1 m/min every minute. Motivation was provided by gently tapping the mouse’s rear [12,13,32]. Time to fatigue was recorded following the third time the mouse could no longer keep pace with the speed of the treadmill. Endurance was determined to be the total amount of time, in seconds, the mouse remained on the treadmill.

### Physical activity

Physical activity was evaluated by assessing voluntary distance ran using a running wheel (Model number: 80820F, Lafayette Instruments, Lafayette, IN). Briefly, mice were individually housed in the wheel running cages for four days. The running distance, in revolutions, was recorded and converted to kilometer. The average distance ran per day was used to score physical activity.

### Frailty criteria

Following the percentiles used by Fried et al. [5], mice that fell in the bottom 20% for walking speed, strength, endurance and physical activity were considered to have a positive frailty marker (i.e., for that give criteria) [12,13]. However, rather than unintentional weight loss, we determined that mice with a high body weight, in which they weighed in the top 20% to be positive for this frailty criterion (i.e., body weight). A detailed rationale for selecting heavy or overweight mice as a positive marker for frailty can be found in Baumann et al. [12]. Criteria were used to identify frailty cut-off values at 23 and 20 months of age for male and female mice, respectively. These ages were selected for several reasons. First, male mice lived 3.7 months longer than female mice (Figure 1). Second, these ages represent a high survival rate; meaning they are near the maximal age before mice begin dying, making it an optimal age to predict frailty. Third, 20 to 23 months for a mouse are equal to approximately 60 to 75 human years [5,10], which correspond to the initial age brackets assessed by Fried et al. [5]. Lastly, because frailty is thought to be reversible [33,34], this age provides adequate time to implement possible life changing interventions.

In our previous publications [12,13], mice were categorized into three distinct groups (e.g., non-frail, pre-frail and frail) based on the number of positive frailty markers they possessed. However, because we did not detect differences in the lifespan of pre-frail and frail female mice, we combined these two groups in the study by Kwak et al. [13]. Therefore, for the purpose of this study, male and female mice were categorized into two distinct groups: mice with two or more positive frailty markers were identified as frail while mice with one or no positive frailty marker were considered non-frail. Table 1 lists cut-off values and the ages (23 and 20 months for male and female mice, respectively) that were used to quantify the prevalence of frailty for all other age groups (i.e., from 17 months until death).

### Statistical analysis

A Kaplan-Meier analysis was performed to assess the sex differences in lifespan. In order to account for variability in lifespan between male and female mice (Figure 1), the prevalence of frailty was also assessed relative to the mean lifespan for each respective sex. Differences in prevalence of frailty, and anthropometrical and functional sex characteristics were examined using an independent t-test. Values are presented as mean ± standard error. Statistical significance was defined as p≤0.05. Data analyses were completed using SPSS 24.0 (IBM Corp, Armonk, NY) or SigmaPlot 14.0 (Systat Software Inc., Point Richmond, CA).
